# Supplementary material for: Piezo-Responsive Hydrogen-Bonded Frameworks Based on Vanillin-Barbiturate Conjugates
Source: Molecules. 2022 Sep 2;27(17):5659. doi: 10.3390/molecules27175659 (PMC9457948; doi:10.3390/molecules27175659)
Supplement: Supplementary file 1 [file molecules-27-05659-s001.zip › Cartesian_coordinates_for_model_structures.pdf]

Coordinates from ORCA-job compound **3a**

O	-11.67212709066238	-5.83231652144865	1.20812095717952
H	-11.36315353540673	-6.75531323849910	1.21854943106860
C	-10.58552645369771	-5.05476412634922	1.09890187446402
C	-10.69832031211735	-3.66256140165706	1.05381365508320
C	-9.29917512615403	-5.65232222864277	1.03107026614491
C	-9.55364808965329	-2.88238755108176	0.94692361214276
C	-8.16018575876351	-4.87853123434719	0.90975918211011
C	-8.26299655680625	-3.46316819328370	0.86316284204519
O	-5.29554289707999	-4.95513008311054	0.55293068525369
H	-9.64915592010546	-1.79476099750379	0.91825691484131
H	-7.17051754028323	-5.32269589377578	0.84850036491879
C	-7.16633187649367	-2.53933304464331	0.73551833566108
C	-4.95181805569588	-3.79015213386111	0.41331688205319
C	-5.80287139729394	-2.59122461228915	0.54919886187698
N	-3.61941383497741	-3.53671153268275	0.08572253928084
C	-2.98879343089557	-2.31611394583213	-0.10772214887279
H	-3.03387237654678	-4.35783637908690	-0.04053915476242
O	-1.82619303280650	-2.21527490115414	-0.42769813331181
H	-7.49742190067829	-1.49542834441852	0.77954455838179
C	-5.16643959054691	-1.24077379754612	0.44841347387235
N	-3.81613010982303	-1.22959334468820	0.11090587689265
O	-5.74998533372957	-0.18720206107423	0.62190003629413
H	-3.38783685493022	-0.31373754693992	0.00784012983897
H	-11.69289590803482	-3.21717748449355	1.10884706047217
O	-9.32944154854510	-7.01248352121996	1.10168901856768
C	-8.10399881293975	-7.71334189122707	0.97928984607433
H	-8.34602491273084	-8.78175848510466	1.04952054122151
H	-7.40303984200576	-7.43820968829229	1.78494801983329
H	-7.62095190059584	-7.50285581574637	0.00965447137390

Coordinates from ORCA-job compound **3b**

O	-11.73027804803553	-5.72893417798498	1.24210679778824

H	-11.43293037239332	-6.65581107043603	1.26490090119454
C	-10.63200769175193	-4.97016091972407	1.11803297056179
C	-10.72268605662365	-3.57777101202557	1.04338384505018
C	-9.35609797836273	-5.59179646140012	1.05984611289766
C	-9.56479486995801	-2.81921013009417	0.92453881516949
C	-8.20288747603686	-4.83829291424118	0.93836347691230
C	-8.28283339218298	-3.42202628909422	0.86458817163198
O	-5.32744933552584	-4.95535298611679	0.65352484423219
H	-9.64262586632428	-1.73134888696334	0.86595656801585
H	-7.21995977184408	-5.29960025365972	0.89966683111891
C	-7.17254610786582	-2.51552140068210	0.72679789437619
C	-4.96980235153577	-3.80048295726220	0.47239546046216
C	-5.80763552371042	-2.58796194254178	0.55889869745313
N	-3.63268733448001	-3.57385875294408	0.14305549906310
C	-2.98488802498806	-2.36742538117551	-0.08041978865845
H	-3.05478889865947	-4.40513870922388	0.05424882991383
O	-1.81828484636532	-2.29072008411169	-0.39289013669574
H	-7.49169791987050	-1.46695409932031	0.73649808988193
C	-5.15302985853455	-1.24899761836161	0.42582813285627
N	-3.79990725112798	-1.26435764693080	0.09891949914037
O	-5.72374349830114	-0.18358990220352	0.56564629543259
H	-3.36006280339673	-0.35724018432515	-0.02978226428753
H	-11.71026401257076	-3.11523996398464	1.08075784122580
O	-9.41549998401133	-6.94990940642648	1.13487618960882
C	-8.21750300400960	-7.70160468382585	0.93986725834571
C	-8.58655645301726	-9.16988440083670	0.89624141298759
H	-7.51059368252433	-7.48225865225074	1.75952037735711
H	-7.73653394628306	-7.37901234282128	-0.00036827989318
H	-9.04766115915616	-9.48986685732037	1.84379190288942
H	-7.68732220792298	-9.78034013906759	0.72115164099351
H	-9.30053027262933	-9.36743977264342	0.08208611297423

Coordinates from ORCA-job **1'**

O	-11.80012343112427	-5.84933883167373	0.65192366216619
H	-11.48341009986417	-6.76966674498072	0.65683656754153
C	-10.71601102525096	-5.06038763530916	0.66953718509637
C	-10.84224784187964	-3.66870162716978	0.68031059012808
C	-9.42144537152772	-5.64433625176427	0.68215222707614
C	-9.70234953126422	-2.87581037362396	0.70903923799554
C	-8.28541080500221	-4.85680189585763	0.71155575790447
C	-8.40311600023249	-3.44252180288599	0.72640461185736
O	-5.38812753841218	-4.86842477553616	0.70489234830981
H	-9.80710148807056	-1.78868949960160	0.71968181967358
H	-7.28740546335509	-5.28691941695425	0.72606030090056
C	-7.31464387407542	-2.50327020173112	0.76503157161955
C	-5.04099898526453	-3.69778103044930	0.70434652565484
C	-5.93803141329235	-2.52264063161839	0.76370453076612
N	-3.67259698443130	-3.41065549795813	0.63850283812137
C	-3.08549364910607	-2.16968742407820	0.61924806053264
H	-3.02382728832138	-4.21168306342861	0.56172301524113
O	-1.88043156312190	-1.98772277932603	0.52315938208984
H	-7.66941315271736	-1.46713552316157	0.80346940858363
C	-5.34052338642824	-1.15563940019084	0.81537546009183
N	-3.95339784211054	-1.10596003488396	0.72332881279700
O	-5.97252531561256	-0.12108691772991	0.91763313057137
H	-3.53009126893037	-0.18198198942210	0.72510382329741
H	-11.84308756894071	-3.23425089828020	0.67024327793525
O	-9.43918865620475	-7.00644231376733	0.66719743669632
C	-8.19986246535450	-7.68705204487063	0.76975435277753
H	-8.43132266575963	-8.76000709439129	0.77532039854211
H	-7.67356559585162	-7.41384570447187	1.70040480799818
H	-7.54214389271135	-7.45295669059955	-0.08381353648972
O	-1.51682031953606	-5.36587687110337	0.36286074875762
C	-0.32684392668600	-5.09431705350713	0.26867033647370
H	5.73343590172918	-8.53779361240334	1.50906254591766

N	0.15098730269168	-3.82089293674436	0.12502019457828
N	0.64612925458506	-6.07942911582800	0.29927118550845
H	-0.55643893076877	-3.06590973074091	0.17409517770766
C	1.48468168950672	-3.43630985032914	0.03556456427365
C	2.03558000996058	-5.92448245113354	0.26568050461817
C	2.50082364718793	-4.53618217483543	0.11013986866755
H	0.31107768187183	-7.03194202234975	0.41319109553476
C	6.17542893098755	-3.68277877982988	-0.07366744412587
C	7.51166170061588	-4.06156868726781	-0.05534563949657
C	3.79813641457416	-4.08588120455133	0.03033252319889
O	1.76667521959168	-2.25934636609189	-0.08502580695248
C	5.13039470683352	-4.63012118685033	0.06575228049427
C	7.84335695553859	-5.40750647369581	0.11095698270612
O	2.72917280161796	-6.92631207315415	0.36246923745228
C	5.47926082167981	-5.99709755639190	0.23761555888650
O	9.12336304052109	-5.80372608127962	0.12513574145259
C	6.81068898402436	-6.36978775075386	0.26845254062296
H	5.91947885410625	-2.62902611261448	-0.20282455920110
H	8.31724807957969	-3.33447463546515	-0.16981464794134
H	3.80024544874505	-2.99633511314353	-0.09184374826029
C	6.35788343021965	-8.69269582105032	0.61242143519268
O	7.28414804269930	-7.63596536349124	0.44443459566457
H	9.12272180593270	-6.76717015659520	0.26160691029517
H	4.67745816291064	-6.72292020059073	0.34876100932317
H	6.95149631513643	-9.60866647306873	0.73096429651509
H	5.69616213836205	-8.79043604942165	-0.26476509334024

Coordinates from ORCA-job **1''**

O	-10.24830424387183	-8.38153680207465	0.57330664888664
H	-9.81031927460244	-9.22190537856494	0.35043572205008
C	-9.31058467482567	-7.42555140021840	0.51648060585535
C	-9.63164698149998	-6.08772099544780	0.76046553510641
C	-7.97452711401669	-7.77738924149034	0.18610661928995

C	-8.63850751283115	-5.11973893507510	0.68173461405704
C	-6.97846706526503	-6.81999848867511	0.14343334326823
C	-7.29307837428021	-5.45629488620969	0.38875323809273
O	-4.13529242214807	-6.41230679132886	0.13834303311012
H	-8.89712490524039	-4.07306455757900	0.85736615111472
H	-5.94712156843919	-7.08368809359018	-0.07308814343301
C	-6.37210004593531	-4.35030504887873	0.33692081500663
C	-3.97249963580565	-5.20154344541939	0.15894038045673
C	-5.01495677021942	-4.16287604112652	0.21085789831384
N	-2.66350735803891	-4.71659241260539	0.14497981900705
C	-2.23948749684094	-3.40333839840192	0.07582041133794
H	-1.92998949103284	-5.41928019419978	0.11507469876585
O	-1.04973452927674	-3.12958244244061	0.00328156524886
H	-6.87743210995222	-3.38123858742870	0.41943932332670
C	-4.60722924975475	-2.72035687774207	0.16781711609284
N	-3.23833403962043	-2.46764785292738	0.09160448856407
O	-5.39552280421734	-1.79478382513721	0.18677153119165
H	-2.92580626529491	-1.48488249948549	0.00599397056673
H	-10.66420521941576	-5.82998655377344	1.00203629940263
O	-7.81557828862778	-9.10502209529625	-0.07288914691976
C	-6.58267397400951	-9.56756934237134	-0.62392627342739
C	-6.78004305819386	-11.00493364524187	-1.06485458324881
H	-5.78426100589139	-9.47881982328675	0.13439012407521
H	-6.29765728726267	-8.92233098737642	-1.47444299872348
H	-7.07922127247550	-11.63660895395091	-0.21402986089189
H	-5.84618920119094	-11.40773574166076	-1.48670729834646
H	-7.56334330949678	-11.07325440628129	-1.83546617152221
H	7.61677754570401	-3.37368027415948	1.04207966560032
H	5.43651653120786	-2.16157965972853	0.65552197582469
O	-2.03012937958779	0.04592004690238	-0.34727884736847
C	-0.88142958816130	0.29122685708465	-0.68533707455266
C	6.67173895283708	1.33630063905515	-2.73685345189520

C	6.84969305825665	-3.78897218189344	0.37017419661954
C	5.32966889998312	1.68608978580861	-2.64282855279821
C	7.12452644388173	0.16742162722502	-2.11859125116199
O	8.41736004199588	-0.18525604710587	-2.15979422995536
N	-0.50984628965097	1.55637883548421	-1.08776667684786
N	0.12214849892005	-0.64378928388132	-0.69808857147289
C	5.95816794298396	-2.68851397816297	-0.16373440670069
C	4.39460244550807	0.86817664353348	-1.96033794972911
C	6.20246068405697	-0.66163830392879	-1.42676649056284
H	7.39110015457513	1.96262199080916	-3.26692483153253
C	0.75269661480446	1.97312402415516	-1.50383506397902
C	3.04839185278688	1.37652165115507	-1.88515035968774
C	1.82061684089049	0.93110812222397	-1.45054673306798
C	1.46744700373814	-0.43606941485700	-1.00865627895602
C	4.86265726100516	-0.32973901292650	-1.35726460453467
H	6.25103245773822	-4.52042591055637	0.93437302722001
O	6.78078952939604	-1.76374539531501	-0.87343712522164
H	-1.24524311485612	2.25818904188216	-1.09329807858264
H	4.98382927015377	2.61321446583984	-3.10548091070571
H	-0.16134003364091	-1.59586182532766	-0.40946456181760
O	0.91771769939924	3.12515778909570	-1.85881176042470
H	8.49821404154610	-1.02008642144480	-1.66573035610460
O	2.23589037200419	-1.38024831922739	-0.91643641922155
H	2.97101431694677	2.40658056462701	-2.25243534518676
H	4.14343320606073	-0.97099167444458	-0.85728392552243
H	7.35760111818708	-4.31524649104182	-0.45332598297423
H	5.18259217090374	-3.08418314559329	-0.84364850037436

Coordinates from ORCA-job **2'**

O	-11.79658207693960	-5.70297646123529	1.76602698467858
H	-11.50142038388263	-6.61769406091806	1.92069371251169
C	-10.70179496953315	-4.97261623216041	1.51754571221580
C	-10.79285752784800	-3.60072964915113	1.26886757902691

C	-9.42750954814926	-5.60324223815303	1.51342559263850
C	-9.63668106562298	-2.87292490637050	1.02182274058647
C	-8.27716047085767	-4.88659971161427	1.23848921055820
C	-8.35964512820173	-3.49013106146269	0.98472803840690
O	-5.41195745831423	-5.05063724855985	0.68221095503826
H	-9.71046256150787	-1.79908093114894	0.83628629250263
H	-7.29847214036791	-5.35938600087775	1.20558295235825
C	-7.26146543019635	-2.60840924272139	0.69903215016680
C	-5.06611804762959	-3.90636071177358	0.42944456188625
C	-5.90399706763103	-2.69508404169297	0.47318782247666
N	-3.74224905022535	-3.68485257545071	0.04256279327168
C	-3.11207922618538	-2.48309013819027	-0.24632474057392
H	-3.15763854918498	-4.51388013742840	-0.02087118392142
O	-1.94633560391326	-2.42615496181627	-0.57935301501576
H	-7.58390422481551	-1.56429634410558	0.62292009893907
C	-5.26176096504014	-1.37701630388289	0.21080494826495
N	-3.92158306440262	-1.37195713707338	-0.11476485131968
O	-5.87933679977919	-0.31772395677234	0.26937521431550
H	-3.49997791585559	-0.44553377018692	-0.31254574847175
H	-11.77641398520024	-3.12837359061963	1.28269970942569
O	-9.49275776099160	-6.93186999597378	1.80680648749156
C	-8.28776028423327	-7.67389876455258	1.85725451370616
H	-7.58845165909720	-7.24611394000501	2.59509718120758
H	-7.79138552747462	-7.69775019164366	0.87230126939085
H	-0.10831384068867	-0.71021271411092	-0.24856683281761
O	-7.00300247969819	3.16075988923169	0.34447404894916
C	-5.84677988626902	3.32549865582256	0.02885586656408
C	1.95849266795040	3.84016892764464	-1.08383933998828
C	0.65827816265043	4.31667744027955	-0.97226994989735
C	2.18239344014249	2.46453072814239	-1.17473112314733
O	3.42269811075723	1.97445514786306	-1.29745612904303
N	-5.30740710235057	4.57637361667571	-0.22867311709178

N -4.94625264742788 2.28238364851420 -0.11865027216047

C 0.43019140138581 -0.72940368497341 -1.20726066449500

C -0.45500309870656 3.43940433440457 -0.92572172022267

C 1.07997638257458 1.56846829812403 -1.14350444015040

H 2.81775285969809 4.51221290167955 -1.11110757796903

C -3.98976150352817 4.88070679157239 -0.54393962088803

C -1.73963884105319 4.07709352661973 -0.80306115791154

C -3.05592919325381 3.71234419608602 -0.62258370971599

C -3.61181884717521 2.35771610906497 -0.45506724146509

C -0.21291906093132 2.04159237462412 -1.01224384419778

O 1.44614773751974 0.26461451467601 -1.25619794551859

H -5.94446476793457 5.36379647097599 -0.14567576380972

H 0.48636530645034 5.39357629613297 -0.91170660990638

H -5.32873163748147 1.33279819651700 0.04384685694581

O -3.66504648475654 6.04022230379243 -0.72395921940238

H 3.33962367366159 1.00543263887364 -1.34633529098671

O -2.97658953180634 1.30564179199866 -0.58313227590915

H -1.66345178644508 5.16970725784333 -0.85010850285577

H -1.06381760372035 1.36856374683198 -0.97519124549578

H -0.30264528126680 -0.59427630256303 -2.01834317709187

H 0.92946918646783 -1.69855086251740 -1.32546151858291

H -8.56217684168320 -8.69335193428508 2.15806453649914

Coordinates from ORCA-job **2''**

O -11.66667677168616 -5.79406683190984 1.70125066819504

H -11.44282831405292 -6.67239231027642 1.34588551673934

C -10.59129764500149 -5.01532604676494 1.52934143788963

C -10.61378869903582 -3.66996424772680 1.90732376495678

C -9.41675135029388 -5.56477903161275 0.94432835845782

C -9.47915086637477 -2.89136180186303 1.72616166084989

C -8.27194374151675 -4.80160617107720 0.80500056701765

C -8.27923791319098 -3.43634864040333 1.20267246668956

O -5.38041544556661 -4.93242953167976 0.56190101702198

H	-9.50047006877770	-1.83827090963762	2.01497369336372
H	-7.35306304850186	-5.22114847425631	0.40466762677620
C	-7.17432590429952	-2.52031518895276	1.12484729203328
C	-4.98856102685812	-3.78530102080229	0.71139561217140
C	-5.81547040582145	-2.58115487896511	0.90336370871035
N	-3.61126055567784	-3.55016321631406	0.73063292292721
C	-2.94237361875934	-2.33564086431430	0.73787789514886
H	-3.02303059012593	-4.37298822262212	0.62941151961819
O	-1.73188851781966	-2.26194235842874	0.67971619563066
H	-7.47913119096284	-1.48597038383186	1.31891416435007
C	-5.14270357980033	-1.25359934751058	0.93400120575605
N	-3.76829214209537	-1.23177152427068	0.80999202874293
O	-5.76181185545059	-0.20063485169179	1.05295844203130
H	-3.32759081723993	-0.29720367395590	0.73649178445807
H	-11.52952659215676	-3.25931843655547	2.33594742854769
O	-9.56349475781747	-6.86584270299219	0.57139123036355
C	-8.49594431121826	-7.52041195978347	-0.11437683012200
C	-8.98416073611531	-8.88798931641008	-0.54970239294363
H	-7.62320929857596	-7.59560671674714	0.55808327591415
H	-8.19011396449715	-6.90851771832829	-0.98209656906358
H	-9.31692176288672	-9.47775713256313	0.31870169766367
H	-8.17403076449563	-9.43747943686223	-1.05287546125036
H	-9.82868458866470	-8.79978258170424	-1.25057956799256
H	2.38592044876291	-1.32565316388849	1.48506322444140
H	0.35901418419880	0.14526401396200	1.23364563425010
O	-7.02396050665702	3.18217781816460	0.50519158986295
C	-5.86819243463686	3.33299903852075	0.18049978303754
C	1.50913511669621	3.28619619571198	-2.63424398851644
C	1.55886186046971	-1.61691607759847	0.81906403721701
C	0.24904977631418	3.81323108598884	-2.38038326271101
C	1.92629455325113	2.14297074247242	-1.94732718684791
O	3.13605754453085	1.60944941812169	-2.16224805008292

N -5.38334101611707 4.51319871679935 -0.36023292042513

N -4.90699920295924 2.34569550848239 0.32410042756272

C 0.75920163514369 -0.41429120402930 0.36976790351189

C -0.64342807571516 3.20472729152921 -1.46029607269767

C 1.05956585441085 1.53147800608547 -1.00117092775076

H 2.19014303778419 3.74437452919123 -3.35350542950898

C -4.10418357416661 4.76039095271797 -0.84357459683307

C -1.91042437970728 3.87268207547883 -1.29622280296647

C -3.12504500467899 3.63802604812465 -0.68942629175135

C -3.56970628536479 2.41058927487856 -0.00579835124104

C -0.20781540760813 2.03895258745774 -0.77066108767741

H 0.89058146812511 -2.30767936461731 1.35304909030577

O 1.61074453412717 0.44329170786070 -0.40041073778613

H -6.05738257193470 5.26623747813927 -0.46722951155999

H -0.06776810946189 4.71513766190968 -2.90863993957518

H -5.24508292705603 1.44752999014223 0.71321281193263

O -3.85175567901147 5.83701407865421 -1.35316912224665

H 3.20160559483207 0.82399273207002 -1.58947463142169

O -2.84716653001084 1.44662437008818 0.26400070596656

H -1.93119744747257 4.84401725958256 -1.80404344926475

H -0.88862689572355 1.56911154979215 -0.06746426089062

H 1.98243508647314 -2.14714350251142 -0.04927146922172

H -0.10056380149918 -0.72063128846710 -0.24468347776458

Coordinates from ORCA-job **3'**

O -10.00411945189480 9.13537684091468 1.03745353603766

H -9.40203176008028 9.89336781933023 1.13809291418309

C -9.24238262619427 8.04033382364162 0.91400854630695

C -9.82042234273075 6.77529299320334 0.77846123014232

C -7.82830868807175 8.16613613602432 0.92830957333096

C -9.00436594769085 5.65922069677713 0.64305567294456

C -7.01593184037248 7.06052432405407 0.76453494979007

C -7.59156951602147 5.77075275648172 0.60957097701886

O	-4.33240679068559	6.21351244135827	0.20865179171558
H	-9.45995700505951	4.67111812413382	0.54616848353455
H	-5.93372117944347	7.14873637003903	0.73085386337912
C	-6.87261833142919	4.53802228759706	0.41749995193717
C	-4.37388483165084	4.99429939551770	0.13867888117527
C	-5.56868329471359	4.13860262215696	0.23134305986182
N	-3.17321279416033	4.30912126839884	-0.05517858934126
C	-2.97691169643745	2.94780314595354	-0.17985718308310
H	-2.33800650262450	4.88419719167852	-0.12399532973788
O	-1.85531218239050	2.48883866479235	-0.35205507711937
H	-7.53637767937980	3.66564877649857	0.40292375051624
C	-5.41079230050786	2.65575080217332	0.07786147294975
N	-4.11059936889229	2.18555935366790	-0.09731753442273
O	-6.33967499797795	1.87157156903675	0.10301328338087
H	-3.97878236550293	1.15895820916720	-0.16799590564772
H	-10.90876712287056	6.69484702860086	0.78844783890339
O	-7.39950030147376	9.44793853175694	1.11014107501738
C	-6.01310157562822	9.66991407831352	1.30163974226224
H	-5.89329440583259	10.73766574390909	1.52725535755949
H	-5.43587308346624	9.41495377806936	0.39725285062870
H	-5.62942594677484	9.07106489208323	2.14494274801373
O	1.00894258731984	0.04131983627709	-0.49701219848833
C	0.06281739720129	-0.70495516980391	-0.40074216009280
H	-0.74986982624409	-7.61194203818839	-0.26957389153573
N	-1.25063424652539	-0.27409262989224	-0.33660964609575
N	0.20568508816014	-2.08584856033707	-0.34453924199781
H	-1.40196347416405	0.75149537599531	-0.36931583926164
C	-2.36733383421478	-1.07005480540513	-0.21963402734579
C	-0.77600575229393	-3.06228973304685	-0.21719597147411
C	-2.15786480028051	-2.54666303789412	-0.13190832110902
H	1.16091095537958	-2.42749376586781	-0.40409059557098
C	-5.08608874359417	-4.92080490278792	0.26746480787898

C	-5.56888197701376	-6.21330309806855	0.44236405894862
C	-3.32277513961218	-3.27005419826815	0.01548480999829
O	-3.48387631779520	-0.55741504287654	-0.17814891989195
C	-3.69592451264269	-4.64854031322244	0.19925583385303
C	-4.67031909470957	-7.27581939764873	0.57582472058858
O	-0.42218277922473	-4.23349491497586	-0.19187537421978
C	-2.79409374191841	-5.73582496376209	0.34110447488506
O	-5.09837033753333	-8.53607309217288	0.73885142592064
C	-3.27554129917565	-7.01430324121878	0.54154948853595
H	-5.79390863184590	-4.09425089246577	0.17382141999607
H	-6.63880505416850	-6.42461618355688	0.48333960932456
H	-4.18947909110435	-2.59953549214867	0.00306119478123
C	-1.10475221760281	-7.98553971757322	0.70585278431946
O	-2.51324995024199	-8.12870121216437	0.71839689122425
H	-4.30676235053032	-9.09705878116155	0.81651697494694
H	-1.72940920542352	-5.53014384949808	0.27533905946803
H	-0.68545578589633	-8.98159701287535	0.89647501345368
H	-0.76905193434347	-7.28802883072142	1.49106168772235

Coordinates from ORCA-job **3''**

O	-9.98693879196368	9.28371270060252	1.22160106246922
H	-9.39396019703098	10.04411953795811	1.08619859459229
C	-9.24291066752375	8.17933478330860	1.07693138021990
C	-9.81641208634111	6.90937676399235	1.16997478209475
C	-7.85351163070474	8.30467256231887	0.80996829396781
C	-9.01234047103712	5.78467395205997	1.03942865730034
C	-7.04538055463682	7.18637480014616	0.71575938654265
C	-7.61312040327843	5.88768371291905	0.83446857798463
O	-4.32995010962863	6.23693691411924	0.68503710344582
H	-9.46458171374473	4.79289342982380	1.10919633406895
H	-5.97376045367909	7.27045750561775	0.55527369604104
C	-6.90920720029091	4.63193443560383	0.77159811624397
C	-4.38987952736828	5.01745967553924	0.71254079941074

C	-5.60701696291117	4.19115047631587	0.71413316055979
N	-3.19383907977268	4.29959231845796	0.75310681710800
C	-3.02070494097816	2.92940944553549	0.75553166998932
H	-2.34040120047753	4.85159761377050	0.76313640126027
O	-1.90114503487985	2.44064683857625	0.78255458079202
H	-7.59097641885832	3.77400654092467	0.79650327369485
C	-5.47402405382384	2.69737379252265	0.69721661363497
N	-4.17467952780431	2.19167193639476	0.72146482765923
O	-6.42111328665840	1.93594505609401	0.67009377029230
H	-4.04984260963829	1.16463623312711	0.71578922794527
H	-10.89057790309947	6.83269976720461	1.34638517554315
O	-7.45307363762989	9.59979436693440	0.67093265352692
C	-6.16424139931318	9.88291181770439	0.12462946988352
O	1.07098297217373	0.05610917619648	0.56765154953186
C	0.12762584401395	-0.70026854573775	0.54537380388547
N	-1.19140666370731	-0.28826115108348	0.62012327453756
N	0.28343107954106	-2.07741785003385	0.44244988991616
H	-1.36040102996429	0.73023522431032	0.70373948662342
C	-2.30673702540478	-1.09684640463106	0.60699767842723
C	-0.69368581208720	-3.06559514152218	0.39755639277542
C	-2.08229970651434	-2.56988218685552	0.47600874645831
H	1.24435505224636	-2.40361339673474	0.38827488035422
C	-5.00173701189398	-4.97361510191172	0.43790706121947
C	-5.50154818020450	-6.27014842233891	0.42154397046874
C	-3.23916377535531	-3.31737051462813	0.45173246823746
O	-3.42613464685712	-0.59837475815821	0.70054411576863
C	-3.60984104834946	-4.70819147447610	0.37682363724458
C	-4.61703082175431	-7.34836810864594	0.33720836419312
O	-0.33424321035827	-4.23100628789633	0.29846913059058
C	-2.72144525645878	-5.81374896686586	0.27023576166037
O	-5.05891820814445	-8.61349741364054	0.33289030258510
C	-3.22130967713756	-7.10187405156493	0.24486044763836

H	-5.69733705704587	-4.13455343710299	0.50755532965015
H	-6.57252759026139	-6.47283647137937	0.47382588890707
H	-4.11236888539567	-2.65954503974693	0.52640858421438
C	-1.10788131977764	-8.16408662838350	-0.21811321340193
O	-2.48791333682996	-8.24487521976918	0.13644218708719
H	-4.27413148462329	-9.18402848687857	0.24953768317054
H	-1.65466312163468	-5.61419784419734	0.21606164410171
C	-0.62168143023555	-9.57198521567621	-0.49952905901665
H	-0.54108456815023	-7.69419416590807	0.60482774481559
H	-0.99608566952107	-7.51395947874600	-1.10448282840613
H	-0.72257137452049	-10.20708830252599	0.39428435126987
H	0.43723277436950	-9.55810889269843	-0.80101240581813
H	-1.20572040264005	-10.02745806808027	-1.31416561697749
C	-6.15564161906076	11.33405671539839	-0.31421532103272
H	-5.97096750017393	9.20737825850117	-0.72755452369041
H	-5.38965307316720	9.68010206776554	0.88523063323032
H	-6.92097183195101	11.51102286980983	-1.08542900367978
H	-5.17404614597182	11.60189867040033	-0.73529254630174
H	-6.36527937411788	12.00108706786376	0.53650508349028

Coordinates from ORCA-job **4'**

O	-5.42780127624762	5.00338489392615	2.11791885399323
H	-5.44926635434755	5.81878681354026	1.58589298128853
C	-4.15551896353050	4.57811231453127	2.14354587681298
C	-3.79515651248952	3.42474808615929	2.84374681138238
C	-3.16905678537226	5.30089557446666	1.42569981303897
C	-2.47650036120743	2.98914280423134	2.81080728072587
C	-1.85488416740047	4.87576019668720	1.40000057304646
C	-1.47785141844873	3.69323850014695	2.09078653241508
O	0.78219499610417	5.46974545527865	0.43625617258425
H	-2.20097118024897	2.07731569702718	3.34457618657659
H	-1.09222851099240	5.41428025227526	0.84526629102747
C	-0.16559873638488	3.10055985797622	2.10306787360554

C	1.48727705407842	4.53118834291356	0.77360895969849
C	1.07640689776491	3.35717105507331	1.56758236250267
N	2.82750429714440	4.55314856274389	0.39079569498892
C	3.81811206115316	3.62101602579686	0.64641119397198
H	3.11582263455361	5.36325488415305	-0.15014440557600
O	4.96840527073157	3.76309710141684	0.30274906345097
H	-0.13927253449382	2.16550898650744	2.67365585634933
C	2.08408614257456	2.28985233068197	1.84605555686048
N	3.36074517501812	2.50987286341596	1.33880136150293
O	1.85523266412324	1.26760284624665	2.46931195987609
H	4.05782768625279	1.80363177759511	1.55851937793994
H	-4.56714249195420	2.88109658779138	3.39024406963736
O	-3.67212384294388	6.38730845281213	0.77627838434249
C	-2.78319661091730	7.23409758211680	0.06436660014803
H	-2.18053709596392	6.65831031997674	-0.65339649798850
H	-2.10617081533760	7.75990363219762	0.75873360973309
H	-0.63852501347158	0.73741082511303	0.76675707929068
O	-7.78905044674317	3.62991502022284	-0.18952653379842
C	-6.63875099972293	3.77258979131865	-0.53290736668445
C	0.97404887153889	3.97088306951683	-2.73175476370663
C	-0.34461101873162	4.40641512363330	-2.69806836284973
C	1.33493740506212	2.81766404704062	-2.03157054594739
O	2.60721433263829	2.39234424194837	-2.00691120309563
N	-6.18168255654613	4.88419034481235	-1.22477356945147
N	-5.64786086483490	2.84068414969531	-0.27756187527181
C	-0.03608284368212	0.16183403928266	0.04864591113010
C	-1.34279921279007	3.70234063138555	-1.97738280723096
C	0.34899468377250	2.09505081077570	-1.31284470812465
H	1.74563736055884	4.51444230749483	-3.27890594165161
C	-4.90499678082618	5.10495583377882	-1.73163259470611
C	-2.65516422994794	4.29479432047697	-1.98919941431318
C	-3.89703001810794	4.03780448372809	-1.45357218419317

C -4.30751965329514 2.86343910046890 -0.65991190959130

C -0.96521261673970 2.52005271003861 -1.28650143662287

O 0.85254135222279 1.00886217271069 -0.66338186426954

H -6.87896546281113 5.59033794003602 -1.44415419956358

H -0.62052481927654 5.31811177758083 -3.23186360877088

H -5.93595551686629 2.03029644729925 0.26307646409861

O -4.67635513992377 6.12766689280846 -2.35421646370941

H 2.62910683979843 1.57705185613338 -1.47473782306729

O -3.60202328450199 1.92522397111035 -0.32247704489900

H -2.68177745961471 5.22995419382062 -2.55959514037137

H -1.72749109058800 1.98154634657375 -0.73123919218406

H -0.71330114865863 -0.36386658021257 -0.64561052331096

H 0.58858427150961 -0.55255172215660 0.59729006877503

H -3.40764216063883 7.94839405587846 -0.48464683984458

Coordinates from ORCA-job **4''**

O -5.44027881491194 5.03056666877940 2.09511697043884

H -5.45935430088214 5.84759456470630 1.56523447545826

C -4.16888484278083 4.60284042400975 2.11840230576762

C -3.80883113481699 3.45035656175166 2.82079784525395

C -3.18375596932604 5.32461002260648 1.39714399620933

C -2.48970506132363 3.01631983016045 2.79137768569390

C -1.87021781672214 4.89670154786216 1.37034418659281

C -1.49207918335516 3.72046502406557 2.07011131405354

O 0.76900504670883 5.50085800062135 0.40253347429200

H -2.21226772443969 2.10762297880270 3.32941943458581

H -1.10814664325560 5.42972469859286 0.80991755513297

C -0.17464803073899 3.14385517961765 2.10065323101457

C 1.47499603541408 4.56826368176233 0.75386130568698

C 1.06967403916898 3.41078415136796 1.57416130748196

N 2.81229167161874 4.57930832298864 0.35744028792591

C 3.80845937333123 3.66122664119150 0.63995806191070

H 3.09533137330688 5.37561130814372 -0.20625235376224

O	4.95701535539758	3.79582177421292	0.28772013053952
H	-0.14019432889253	2.21868354427684	2.68681905022042
C	2.08856297034753	2.37051819994116	1.89883622298352
N	3.35978420733743	2.57348828474628	1.37480571233859
O	1.87014190464884	1.37944094285727	2.57545303457469
H	4.06317727675442	1.88330287493214	1.62342157997233
H	-4.58059282607940	2.90830640010083	3.36924266798628
O	-3.68045896232108	6.41569669805243	0.75401599818648
C	-2.76766576562302	7.29907996084108	0.09440287365940
C	-3.55498534621354	8.45085686239635	-0.48980703297857
H	-2.23104237521487	6.74084612982457	-0.69028691418424
H	-2.01756497523723	7.64222179692763	0.82837953100176
H	-4.27638791779528	8.08085796863097	-1.23206197987252
H	-2.86879329464274	9.15103873924454	-0.99159326694073
H	-4.09061862508481	8.99946903420924	0.30139977982173
H	1.44955598495803	-0.68690993966129	1.34839010619357
H	-0.59636486818693	0.65190415952794	0.80383765747807
O	-7.78174586152286	3.59966940425163	-0.17234214542441
C	-6.63322139151721	3.73377807271023	-0.52486098593080
C	0.98448790095470	3.94304626344439	-2.70451840353498
C	0.72888093460327	-1.05759188931363	0.60575962956463
C	-0.33465007085053	4.37708454188526	-2.67577331626841
C	1.34411059654235	2.79039117100484	-2.00219450979228
O	2.61550289825677	2.36267812018740	-1.97822136056813
N	-6.18443295201716	4.82087684401434	-1.26055883445169
N	-5.63718954949545	2.81570645978374	-0.24184476397628
C	-0.05828989910424	0.09358576278566	0.02020348073890
C	-1.33269050582314	3.67282297365333	-1.95517186870465
C	0.35854134084123	2.06839950971162	-1.28177165826895
H	1.75654839587583	4.48517497581656	-3.25245986917806
C	-4.91313442014811	5.02339927375998	-1.78459508805467
C	-2.65006307651833	4.24954284407201	-1.98609320271512

C	-3.89447593823228	3.98297920950322	-1.45960943632429
C	-4.30004093927832	2.82598869982465	-0.63874973086327
C	-0.95500605325441	2.49631848397506	-1.25561847643778
H	0.04247453145640	-1.75774271475682	1.10730765383366
O	0.85486687925747	0.97716561689422	-0.63862530411942
H	-6.88766673135170	5.51109635245125	-1.50948727954924
H	-0.61171766466545	5.28593629474249	-3.21374479870833
H	-5.92033550502768	2.01980432394608	0.32234834848015
O	-4.69444888221421	6.01424256101003	-2.46148137971849
H	2.63429801266177	1.54552499016660	-1.44850017690854
O	-3.59434567594906	1.89322271496707	-0.28722836227102
H	-2.68427352234427	5.17455375374798	-2.57251392960585
H	-1.71741073003488	1.96317323942834	-0.69578427407174
H	1.26538515398985	-1.60645820482506	-0.18466370158297
H	-0.80730370623785	-0.25024268693507	-0.71458249030522

Coordinates from ORCA-job **5'**

O	2.35384430422268	-4.51373442991861	3.95988557013763
H	1.94421905372759	-5.39695849978992	3.98590464145761
C	3.63546611115159	-4.65688875785691	3.59081451834468
C	4.45757366130090	-3.54147155940797	3.41044017701314
C	4.16026337893778	-5.95435104438016	3.35449834633466
C	5.77308005907968	-3.71565640897832	3.00043478417238
C	5.46921565166969	-6.12972889271459	2.94242769754687
C	6.31314017127560	-5.00355409880489	2.75190571141796
O	7.39992409474039	-8.05334352082207	2.04195120000836
H	6.40295825557490	-2.83672471658507	2.84924170200041
H	5.87195999086330	-7.11616728461335	2.73109033094204
C	7.68444638888023	-5.02876376672979	2.30814487426482
C	8.40605479451612	-7.41694636632562	1.75947689024882
C	8.60718493902646	-5.96463605112241	1.89588146504986
N	9.48486160884426	-8.11942344268638	1.22237777354094
C	10.65686843848986	-7.61265582431234	0.68857916058629

H	9.32541921650117	-9.10882432134669	1.05815222607526
O	11.43333734070588	-8.29159316104099	0.05110931983003
H	8.12551953031514	-4.02655611409840	2.26847355317485
C	9.92295249644103	-5.37844451187788	1.49165558004002
N	10.84838773135025	-6.27204635734700	0.95185541227943
O	10.21251286698424	-4.19968683827817	1.57561045911634
H	11.68502443835252	-5.85695116972229	0.55114362548154
H	4.03767512335178	-2.54919191332927	3.57973504446787
O	3.25043359768002	-6.94737409093671	3.55718421498974
C	3.65826219864007	-8.28955580081986	3.34010743440430
H	2.79100985565779	-8.91980389330109	3.57403483624539
H	4.49630311753243	-8.55912346489530	4.00403286607794
H	3.97009718721727	-8.44516333684422	2.29387940874864
O	0.38817171170665	-5.86506119327816	1.67767359227843
C	1.43128925465583	-5.58200763074814	1.12868061574433
H	7.22285696802440	-9.12234859329367	-0.49619941905718
N	1.86119416876299	-4.28490660113320	0.93404532131707
N	2.30763371485130	-6.53542234183445	0.63761680888521
H	1.25805751952505	-3.55754478423922	1.30740700732989
C	3.08478933930019	-3.86552561479490	0.41953780127628
C	3.59407169060946	-6.35202720773767	0.12916761984751
C	4.02714219630050	-4.95054921096878	0.00958308234825
H	2.01480674382116	-7.50031424739100	0.75755445308730
C	7.53061664749259	-4.06231502210483	-1.07401866310680
C	8.80359729974415	-4.43355252954392	-1.48306823197502
C	5.25389720369252	-4.48194575609395	-0.39963636518441
O	3.33231007906219	-2.67573201570404	0.35523283274183
C	6.51022638448974	-5.02054621237291	-0.85037733123714
C	9.09336219890259	-5.78293177117152	-1.69588216235477
O	4.24268930177606	-7.35072028237691	-0.15207963587772
C	6.81392619885743	-6.38980409889910	-1.07067241147344
O	10.32306315403674	-6.16546037944401	-2.06542441460765

C	8.08031423785990	-6.75664391964891	-1.49127481386807
H	7.31043729503223	-3.00609737986545	-0.90639075803630
H	9.59608332434864	-3.69860919257177	-1.62851159292753
H	5.26882012143927	-3.38754955101249	-0.34957311813274
C	7.60262899349071	-9.10045560135803	-1.53180616150191
O	8.50754532451523	-8.02750114923223	-1.72622825434182
H	10.35191126609046	-7.13875045806422	-2.09183318066972
H	6.03941718076594	-7.12756531059067	-0.87969817968717
H	8.16684484455766	-10.01739000515551	-1.74748729328054
H	6.74459003325604	-9.02322230048243	-2.22023597153427

Coordinates from ORCA-job **5''**

O	2.46560653766174	-4.59632964521036	4.05848956306289
H	2.05696649007112	-5.48019354231941	4.06536848590114
C	3.74104353823397	-4.72781250193875	3.66301720740764
C	4.56320335629952	-3.60655305229976	3.51395831460560
C	4.25540878394352	-6.01455707760617	3.35993846336558
C	5.86886742357382	-3.76313197471882	3.06642651241557
C	5.55956646262906	-6.17184127702676	2.92865661171859
C	6.40055470008046	-5.04203253728875	2.75944109152852
O	7.44854622424387	-8.09143637662963	2.01144442233931
H	6.49500350551090	-2.87898795755427	2.93053522464418
H	5.95820671989610	-7.14984190043090	2.68065835180988
C	7.75328767177513	-5.06304080225123	2.26372381029008
C	8.44008384070185	-7.45387067271347	1.68063910322742
C	8.65020983064876	-6.00256752716576	1.80213891041994
N	9.48867703551598	-8.16269890733333	1.09826595500354
C	10.62761443879353	-7.66373810124676	0.49111377925852
H	9.32150119167932	-9.15535372074364	0.96427854444866
O	11.36377290195888	-8.35257845412627	-0.18347930144234
H	8.19639072885756	-4.06233389253263	2.21215921934448
C	9.94444566963517	-5.42344242535193	1.32500644067040
N	10.83242819010062	-6.32078666841310	0.72743752045863

O	10.24463634621888	-4.24668050016500	1.39385441532219
H	11.64561813832773	-5.90900433867328	0.27796387740280
H	4.14832085150736	-2.62202595956612	3.73349063136559
O	3.34990629271520	-7.01830303988690	3.51078591121412
C	3.74884963760191	-8.34636925576006	3.15200398630069
O	0.29572426955314	-5.68590680502019	1.82598575411630
C	1.33896700152078	-5.42014543166504	1.26874454484926
N	1.80666608300611	-4.13357219509475	1.09233643118789
N	2.17847766840492	-6.39022210664698	0.74764585299151
H	1.23202747642075	-3.39433334433287	1.48655889385754
C	3.04065560135614	-3.74480697103494	0.57647585724709
C	3.46500596012111	-6.23934596675683	0.23442253239193
C	3.94200249760117	-4.85190469481057	0.13404015235613
H	1.86032507261115	-7.34779122105615	0.86063291159929
C	7.42245310080897	-4.06042886618533	-1.09245871210659
C	8.65516147228977	-4.46555868736130	-1.58436485936370
C	5.17381543361347	-4.41713473691613	-0.29813779795360
O	3.32687628837046	-2.56269313482005	0.53313490384066
C	6.39258833210042	-4.99105683612913	-0.80410730098180
C	8.89487219306439	-5.82272325008108	-1.80875305473232
O	4.07951787951027	-7.25574988410803	-0.06377157608242
C	6.64903481657420	-6.36936248914564	-1.02848552554715
O	10.08559585628087	-6.24083405554976	-2.25853226025029
C	7.87706602751388	-6.77107101447036	-1.52317404560608
H	7.24191628527013	-2.99879238949901	-0.91222142071735
H	9.45558596481903	-3.75213212619906	-1.78348910429668
H	5.22762610187583	-3.32494201304566	-0.23102612138914
C	7.41073902964828	-9.12860132186237	-1.36600943543504
O	8.26182947426483	-8.05227202247255	-1.76292929702544
H	10.08323075814982	-7.21496485067307	-2.27671655157815
H	5.86968417208157	-7.08639135405619	-0.78706215217957
C	2.55041089293545	-9.25885560790454	3.28203385689975

H	4.57279525133203	-8.66541062038150	3.81381412010632
H	4.13055867840530	-8.34107111701152	2.11792752093171
H	2.18228817703758	-9.29877740447962	4.31869561125642
H	2.83046668304966	-10.27675878984234	2.96849997692394
H	1.72153955825838	-8.91447141706404	2.64396517111300
C	8.18654058576870	-10.41660736979188	-1.55123471051511
H	6.49461003231986	-9.11475007229876	-1.98213499545470
H	7.10542269827125	-8.98651674440275	-0.31542514823094
H	8.44020908520499	-10.57828258677229	-2.61029797535672
H	7.59051641228945	-11.27207351673380	-1.19851143747893
H	9.13240062011317	-10.38613487537007	-0.98746766147076

Coordinates from ORCA-job **6'**

O	6.61233133584333	0.03156826333957	0.14305497580256
H	7.35550732340998	-0.38464171630293	0.61531883446095
C	5.49846352851083	-0.59805826657667	0.54094264314418
C	4.24540659604417	-0.24402792863190	0.03548340900944
C	5.60220338843423	-1.65460646280305	1.48372614286881
C	3.12148339894102	-0.95666550444050	0.43515318787317
C	4.49085575346679	-2.37757842422312	1.86984754647751
C	3.21743152163935	-2.05300810070932	1.33112492314399
O	3.65744582094968	-4.91183815323757	2.97096970884779
H	2.15099037195426	-0.70460693758900	0.00293441629587
H	4.56572733773968	-3.22243836449388	2.54779947820244
C	1.99906921454950	-2.78870711701732	1.53303633588927
C	2.47332966394427	-5.02980084978237	2.67657963579750
C	1.61658621659294	-4.00497386594195	2.05657814334552
N	1.85486421143756	-6.24855728981087	2.93778304762286
C	0.55283534755153	-6.63360438138750	2.66012109813696
H	2.45304351799554	-6.97902863374254	3.31226333946660
O	0.14228019710726	-7.75682988860696	2.84410386937911
H	1.13485414614855	-2.28594804097299	1.08568086715327
C	0.17643613576391	-4.32063914921838	1.82481856592803

N	-0.23198283625889	-5.60495370098377	2.16726572950031
O	-0.62519472525474	-3.54942853780749	1.32602832998481
H	-1.19963476981228	-5.83812401896371	1.95793907647614
H	4.17832122371605	0.57297811228419	-0.68472314250183
O	6.88107118083644	-1.86841680178350	1.90100415237457
C	7.11508719446607	-2.86162963152036	2.88338436427156
H	8.18502670692841	-2.81603536469004	3.12309414644157
H	6.86648499698751	-3.86475634674950	2.50241106304027
H	6.52464977985004	-2.66150766031878	3.79329642654709
O	-1.87886388092483	-5.39717996828536	-1.52486071685636
C	-0.74044290402405	-4.99069806783825	-1.51052242788143
H	5.16925104662871	-6.39158793999692	2.14173541980920
N	-0.36270930547943	-3.74261285732454	-1.97170588273116
N	0.31895342968020	-5.74639666007416	-1.02608900562990
H	-1.11915533439167	-3.13333172654595	-2.27004780091839
C	0.90602422530397	-3.18011992812782	-1.93407115102002
C	1.64759730213652	-5.37011267317946	-0.85790013776033
C	1.99934897193411	-4.05905579098548	-1.42347850290391
H	0.06965848805477	-6.65940071446372	-0.65522377085918
C	5.60207473996332	-2.95419109315122	-1.65912816970559
C	6.93642746501180	-3.09717615671883	-1.30050253751131
C	3.25433696137157	-3.51040646637952	-1.55929748627522
O	1.07361982499240	-2.02745954294822	-2.29608687989070
C	4.60134129325606	-3.83202299432935	-1.17401255321338
C	7.30074645551375	-4.11185851364232	-0.41257617608950
O	2.39195029058527	-6.14228719585427	-0.26640824543730
C	4.98790901493156	-4.87391483696146	-0.29061547204003
O	8.57034364509568	-4.25884969985216	-0.00434597518141
C	6.31139675579231	-4.99847793459064	0.08498377107265
H	5.31144891305689	-2.13698943677049	-2.32136837570005
H	7.70427021022218	-2.41788599565711	-1.67254936183789
H	3.19294116896652	-2.55545961604889	-2.09032751791303

C 5.92113796603177 -6.88530566542029 1.50648366372477

O 6.81239656491296 -5.92352284840810 0.95397353731114

H 8.59038029949851 -5.04052006525491 0.57559668101608

H 4.22345102708091 -5.54546286601281 0.08725924076688

H 6.53815223731674 -7.56570689627541 2.10801465881244

H 5.41401934799710 -7.45590108621865 0.71008085986239

Coordinates from ORCA-job **6''**

O 6.74496555971536 -0.05869550300725 0.38167234999165

H 7.45994614563646 -0.51600995440768 0.85973519011699

C 5.60019240122477 -0.66420158432057 0.72707668202016

C 4.37425922248447 -0.25772693822462 0.19330996452124

C 5.64361125540687 -1.75033323292478 1.64053118933855

C 3.21673470039166 -0.94711809889290 0.53447931625028

C 4.49453601984593 -2.43395555256948 1.98547401678195

C 3.25014840415054 -2.06168774231173 1.41240562513959

O 3.56659737837566 -4.92725021354830 3.06986445368522

H 2.26921943897638 -0.65952369069053 0.07362958118840

H 4.51799819977752 -3.28962387696615 2.65227358648091

C 2.01077725821802 -2.77627625024198 1.55418747544139

C 2.39015158255111 -5.01732398831348 2.73694570763316

C 1.58426420076670 -3.98563050579908 2.06069268643965

N 1.72782377083659 -6.21025352666447 3.01210253767009

C 0.43923929087057 -6.58155548629425 2.66561497929591

H 2.29478758320668 -6.94408081237304 3.42656686678442

O -0.00202076877091 -7.69279285517671 2.85182606265691

H 1.17936385793748 -2.26253369479425 1.05987756512405

C 0.16097656983179 -4.29232324303180 1.73399982244454

N -0.29504249199599 -5.55453861409813 2.09645697756066

O -0.58745827722807 -3.53539524943319 1.13824872143988

H -1.24841884506307 -5.78098171735822 1.82415956823008

H 4.35665739276831 0.57950852893662 -0.50641058221290

O 6.90229678553775 -2.03674547338209 2.07130747757744

C	7.07849693032703	-3.07876102069126	3.03129225702833
O	-1.83507756708778	-5.51827294430189	-1.74118446012263
C	-0.71052678236199	-5.08344811402876	-1.65030631215844
N	-0.33343659162905	-3.83165432804526	-2.09942515737400
N	0.32926348206329	-5.80756886836220	-1.07967737133227
H	-1.08313280391478	-3.24404655327763	-2.45310145131418
C	0.91139866839040	-3.23130577951588	-1.96423082834640
C	1.63045906259456	-5.39247710816256	-0.81628728537210
C	1.99179574201685	-4.08468998581459	-1.38391756957464
H	0.07148898903177	-6.71591852598381	-0.70292730846261
C	5.59164767393342	-2.97434246556878	-1.56845820024998
C	6.93118962256469	-3.14184915989942	-1.23920559673516
C	3.24389093225371	-3.52298096343134	-1.47700260581477
O	1.07437584070937	-2.07547337468805	-2.31539454145916
C	4.58767171136891	-3.84343873678558	-1.07570635396932
C	7.29646773643975	-4.17623411070695	-0.37681707732232
O	2.34481138876007	-6.12555155441590	-0.14272925367346
C	4.97162010269311	-4.89124699679882	-0.19795556542178
O	8.57697996770721	-4.36889981093657	-0.01717052393101
C	6.30170338831187	-5.04102567243524	0.14712441263069
H	5.30078832014849	-2.15005011571954	-2.22158925695400
H	7.70235546666626	-2.47294277813407	-1.62282582922599
H	3.18898202746757	-2.56855363738726	-2.00998049966313
C	5.92267934838954	-6.91336638842712	1.61672274637779
O	6.81025871094870	-5.97493085307785	0.99925695287242
H	8.59045550975467	-5.15726905402809	0.55456164692857
H	4.20271888954562	-5.54747351813186	0.19767340291576
C	6.76548424055969	-7.87049681127015	2.43335734506353
H	5.35469423800524	-7.44529962390169	0.83289637095488
H	5.20347540273190	-6.35987456310894	2.24305727875251
H	7.45657157720251	-8.43665213399396	1.78874580219890
H	6.11925433984939	-8.58781116456014	2.96264375689151

H	7.35889860525393	-7.32319033726041	3.18260230859001
C	8.56227121501548	-3.24623378425787	3.28039672810006
H	6.63953590101572	-4.00976696367116	2.63871822503954
H	6.53419592497004	-2.80895226149427	3.95432131008167
H	9.07306551204320	-3.51908324367679	2.34543589244073
H	8.73566236202380	-4.04204711142018	4.02081524022678
H	9.00722827878230	-2.31587030673910	3.66586354978323
