# Supplementary material for: Piezo-Responsive Hydrogen-Bonded Frameworks Based on Vanillin-Barbiturate Conjugates
Source: Molecules. 2022 Sep 2;27(17):5659. doi: 10.3390/molecules27175659 (PMC9457948; doi:10.3390/molecules27175659)
Supplement: Supplementary file 1 [file molecules-27-05659-s001.zip › Supplementary data.pdf]

Supporting Information

for

Piezo-responsive hydrogen-bonded frameworks based on vanillin–barbiturate conjugates

By

Anna S. Nebalueva,[a] Alexandra A. Timralieva,[a] Roman V. Sadovnichii,[a] Alexander S. Novikov,[a] Mikhail S. Zhukov,[a] Aleksandr S. Aglikov,[a] Anton A. Muravev,[a] Tatiana V. Sviridova,[b] Vadim P. Boyarskiy,[c] Andrei L. Kholkin,[d] and Ekaterina V. Skorb*[a]

Table of contents

Figure S1. ^{1}H NMR spectrum of compound **3a** (DMSO-d_6, 400 MHz, 298 K). 2

Figure S2. ^{13}C NMR spectrum of compound **3a** (DMSO-d_6, 101 MHz, 298 K). 2

Figure S3. ^{1}H NMR spectrum of compound **3b** (DMSO-d_6, 400 MHz, 298 K). 3

Figure S4. ^{13}C NMR spectrum of compound **3b** (DMSO-d_6, 101 MHz, 298 K). 3

Figure S5 Arrangement of the drops of precursors transferred onto the glass slide. 4

Figure S6. Optical microscopy images in polarized light of compound **3a** (left) and compound **3b** (right) 4

Figure S7. Force–distance curve of compound **3a** (left) and **3b** (right) 4

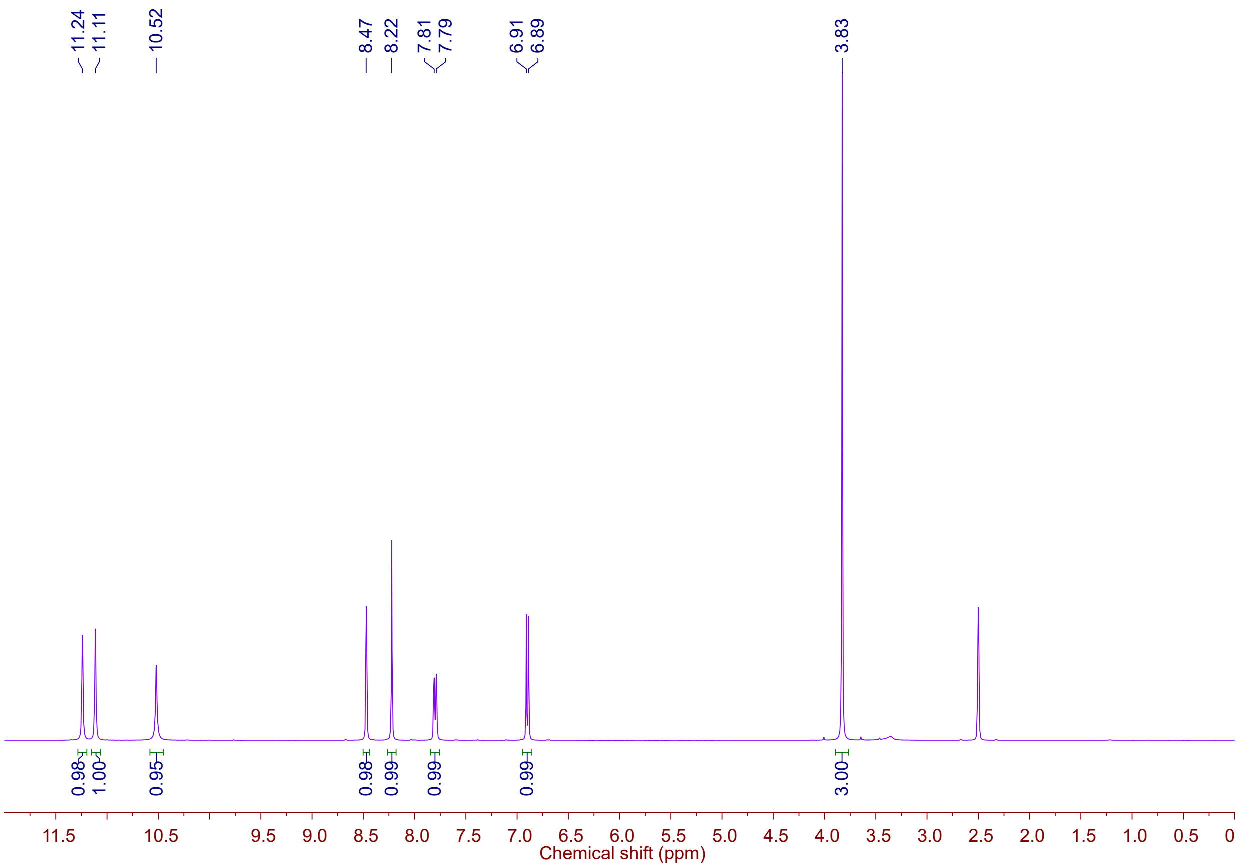

Figure S1. ^{1}H NMR spectrum of compound **3a** (DMSO-d$_6$, 400 MHz, 298 K).

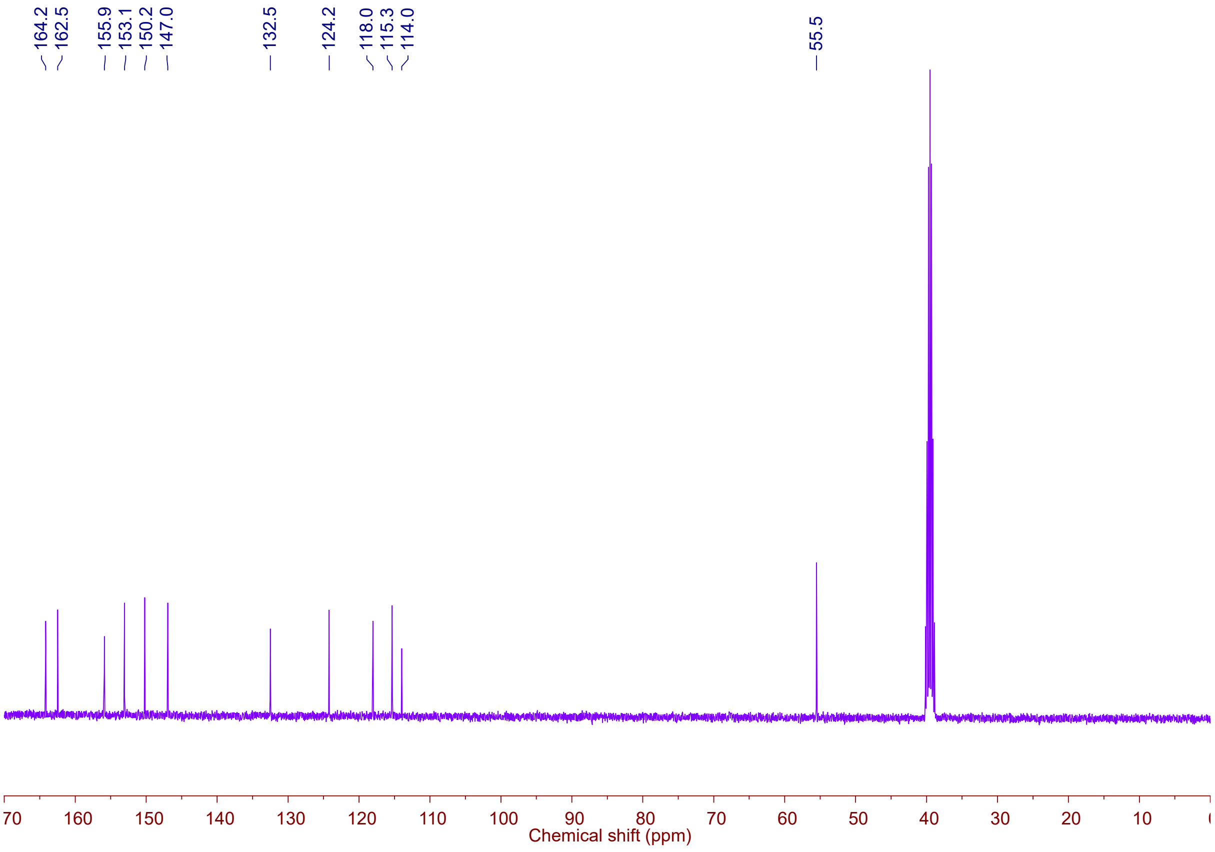

Figure S2. ^{13}C NMR spectrum of compound **3a** (DMSO-d$_6$, 101 MHz, 298 K).

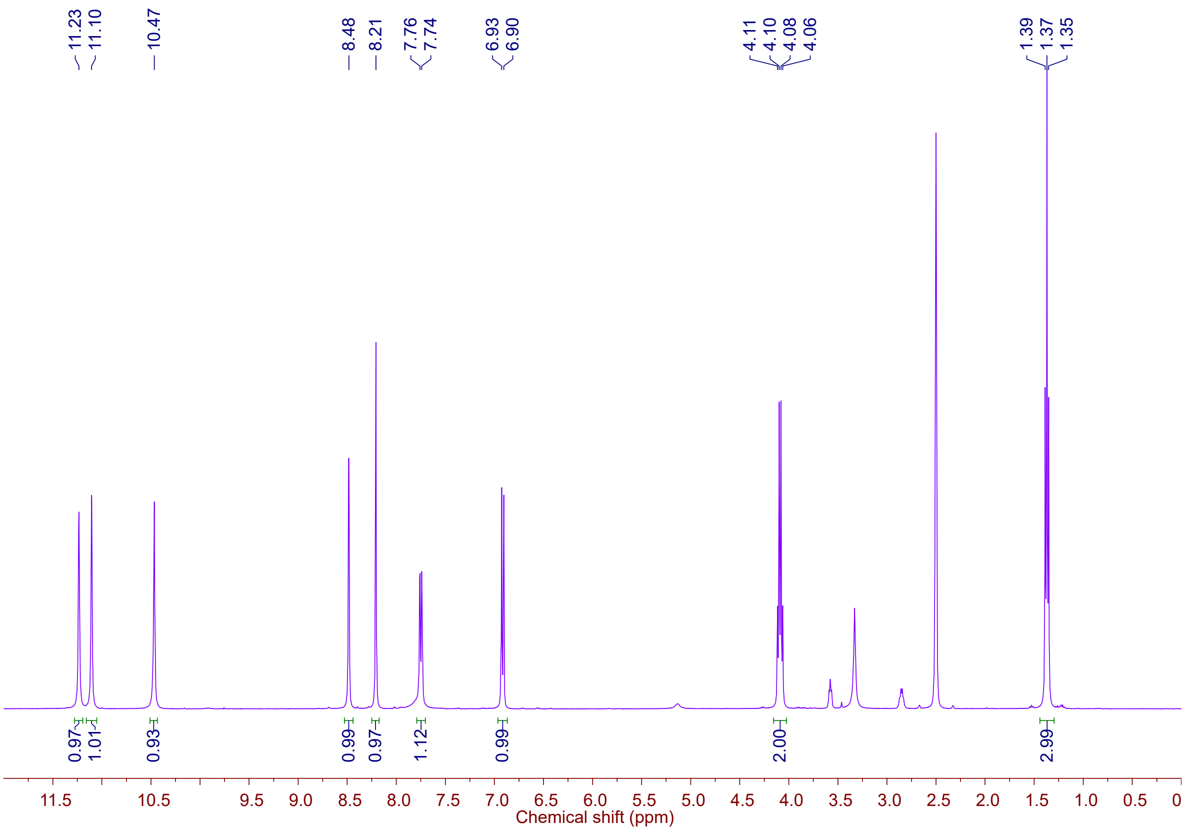

Figure S3. ^{1}H NMR spectrum of compound **3b** (DMSO-d_6, 400 MHz, 298 K).

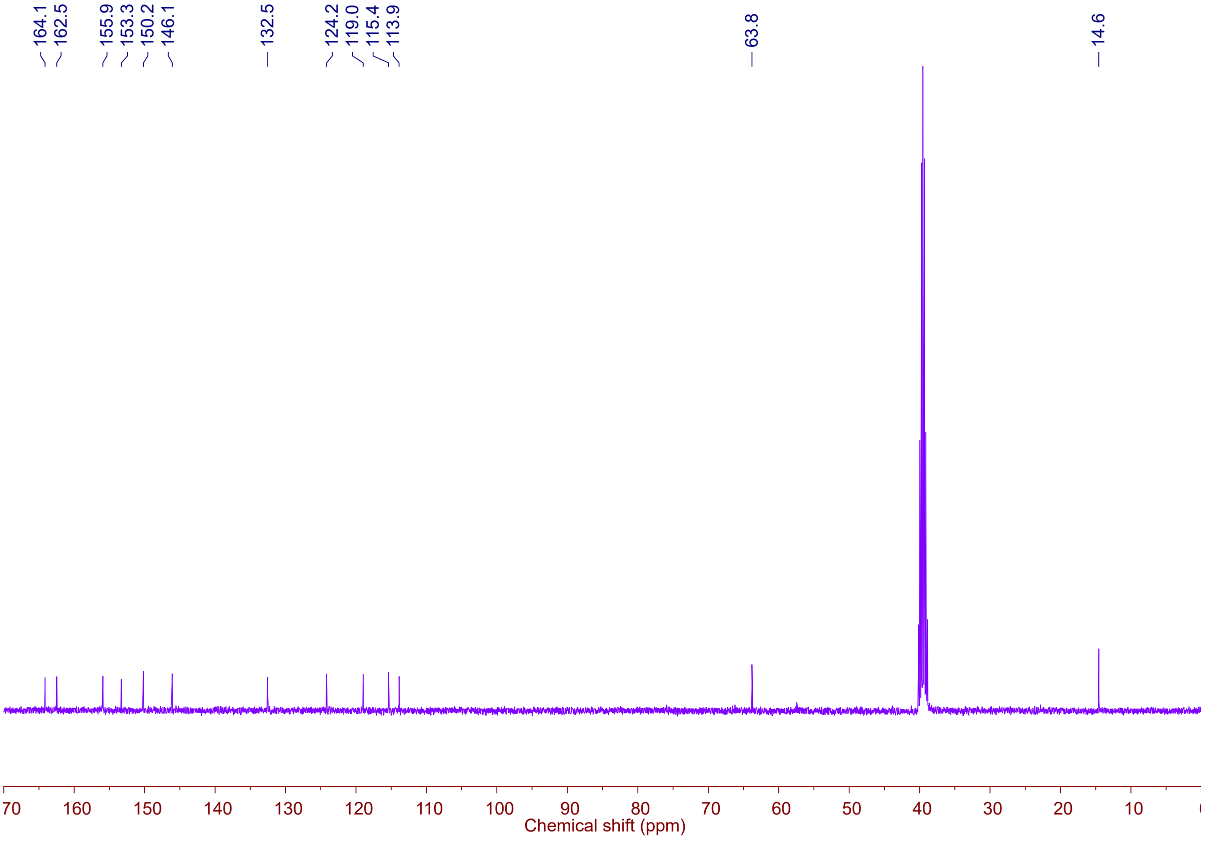

Figure S4. ^{13}C NMR spectrum of compound **3b** (DMSO-d_6, 101 MHz, 298 K).

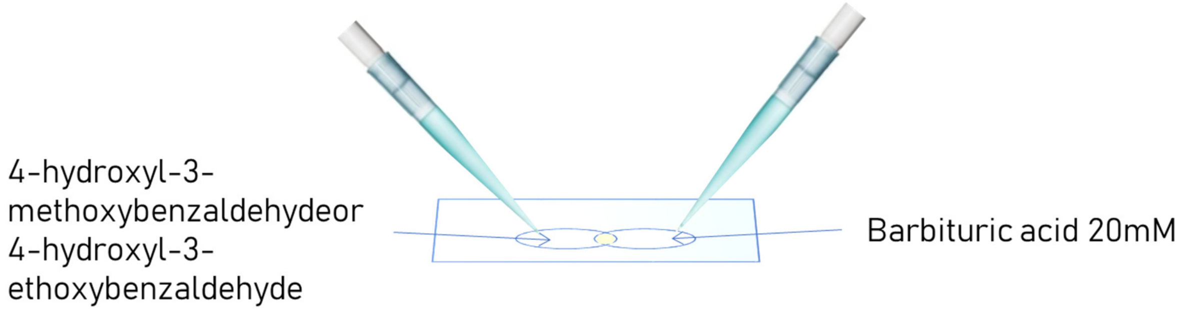

Figure S5 Arrangement of the drops of precursors transferred onto the glass slide.

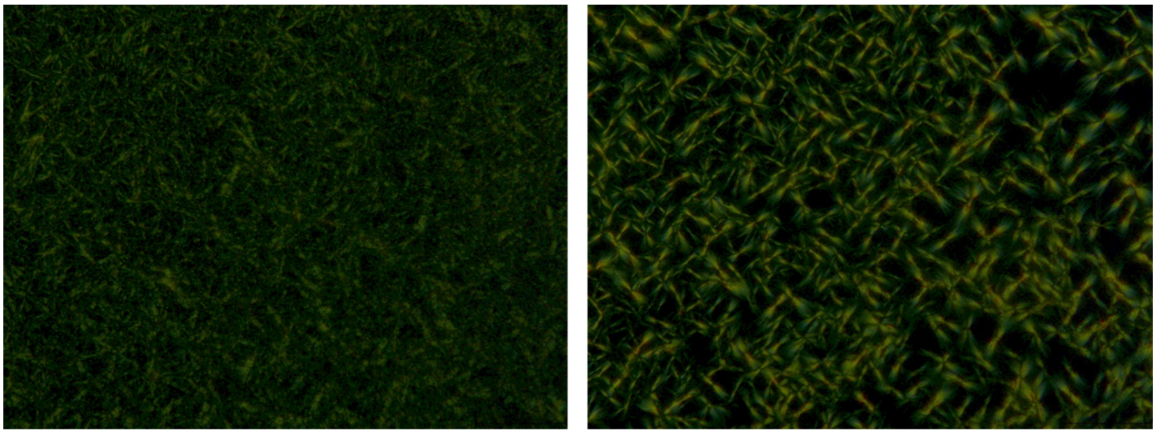

Figure S6. Optical microscopy images in polarized light of compound **3a** (left) and compound **3b** (right).

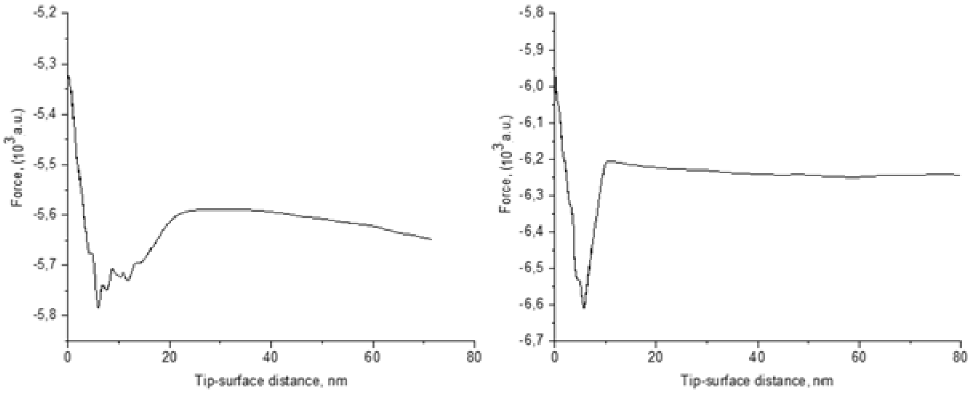

Figure S7. Force–distance curve of compound **3a** (left) and **3b** (right)
